# News Items

**Published:** 1918-10

**Authors:** 


### NEWS ITEMS.

The Henry Ford hospital has been taken over by the United States government. It is to be used for the care of wounded soldiers, and will have accommodations for 1600 patients.

Instructions were recently issued by the War Department to increase the facilities at the base hospital at Fort Sam Houston, at an estimated cost of $258,000. All buildings are to be equipped for summer and winter use.

Narcotics valued at $15,000, found in three drug stores in Memphis, Tenn., were confiscated in a raid conducted by United States Marshal Stanley Trevezant and his deputies after examination of the druggists' books.

Denial of recently published reports that the nursing needs of the Army has been met is made by Brig. Gen. Charles Richard, acting surgeon general, who stated that 25,000 nurses must be obtained before the end of the year.

Two San Antonio physicians were each fined $10 in the police court recently. They were charged with failing to report typhoid fever cases. The arrests were made by the police on affidavits filed against them by Dr. W. A. King, City Health Officer.

Dean W. F. Carter, of Medical Department of the State University, said he did not believe the attendance in the medical college would be affected by the war as it will be in the other college because all the men attending the medical college are counted members of the medical reserve corps of the army or navy and are not affected by the draft. Most of the men of the higher classes are expected back because they have been requested by the government to finish thir courses at which time they will become officers in the United States medical corps, provided they serve their year's internship as required by the government. In regard to the pharmacists, Dean Carter stated that no provision had been made for them and it is not known just how many will return.

Dr. Edward H. Cary, dean of Baylor College of Medicine and Dentistry, announces that a Pasteur Institute will be organized immediately in connection with the college and with the Texas Baptist Memorial Sanitarium. That department of the institution will be established to meet the demands of that section of the State, obviating the necessity of going to Austin for treatment.

The direcors of the sanitarium have had the matter under consideration for some time, and the situation at present is considered so strong from a scientific standpoint that the Pasteur Institute will be organized as a fully recognized department of the medical college. Dr. T. P. Goodman has been added to the faculty of the college and will have charge of the new department.

Dr. Crile, just back from a base hospital at the front, says that the Central Powers are not nearly exhausted, that the war is not half over, that German prisoners are found to be well nourished, clothed and armed. He says there is no glimmer of dawn on the war's horizon.

A convalescent home for American nurses, the first of its kind in England, has been opened by the American Red Cross at Putney.

The convalescent home will be under direct charge of Miss Carrie Hall, chief nurse for the American Red Cross in Great Britain.

A "model system and a model corps" were the comments in a report to Washington made recently by a dental inspector who went to Camp Travis for the purpose of inspecting the dental work in the camp.

At Camp Travis there are 39 dental surgeons and 40 assistants with Col. A. Carpenter, camp dental surgeon. Three buildings are utilized in the work. As soon as a man comes into camp, in fact, as soon as he goes into quarantine, he is given a dental examination and his teeth put into perfect condition. Unless this is done, he cannot go over seas.

In less than six months the medical department of the United States army has established sixteen model sanitary trains which are now running on the French railroads and are destined for the American army. Seven of these trains are side-tracked in Paris for emergencies. One train has 360 beds and can take care of more than 640 wounded. Each coach has a bath room, lighted by electricity and has telephone connections.

Following a favorable report made by Dr. J. F. Christianson of Salt Lake City and Dr. Alexander H. Reynolds of Philadelphia, representing the National Dental Educational Council of America, Government recognition of the Baylor University College of Dentistry is assured. This recognition will put the students of the college on the same basis with those in the medical colleges as members of the Medical Enlisted Corps.

